# Analysis of the Binding Sites of Porcine Sialoadhesin Receptor with PRRSV

**DOI:** 10.3390/ijms141223955

**Published:** 2013-12-09

**Authors:** Yibo Jiang, Faheem Ahmed Khan, Nuruliarizki Shinta Pandupuspitasari, Ishwari Kadariya, Zhangrui Cheng, Yuwei Ren, Xing Chen, Ao Zhou, Liguo Yang, Dexin Kong, Shujun Zhang

**Affiliations:** 1 Key Laboratory of Agricultural Animal Genetics, Breeding and Reproduction of the Ministry of Education, Huazhong Agricultural University, Wuhan 430070, Hubei, China; E-Mails: gytjyb@webmail.hzau.edu.cn (Y.J.); faheemgenetics@gmail.com (F.A.K.); shintse@gmail.com (N.S.P.); Ipkhau34@gmail.com (I.K.); Yuweiren882@gmail.com (Y.R.); Xincheng41@gmail.com (X.C.); Zhouao6@gmail.com (A.Z.); ylg@mail.hzau.edu.cn (L.Y.); 2 Royal Veterinary College, Hawkshead Lane, North Mymms, Hatfield, Hertfordshire AL9 7TA, UK; E-Mail: zhangruicheng0@gmail.com

**Keywords:** sialic acid, binding site, Sialoadhesin, porcine, PRRSV

## Abstract

Porcine reproductive and respiratory syndrome virus (PRRSV) can infect pigs and cause enormous economic losses to the pig industry worldwide. Porcine sialoadhesin (pSN) and CD163 have been identified as key viral receptors on porcine alveolar macrophages (PAM), a main target cell infected by PRRSV. In this study, the protein structures of amino acids 1–119 from the pSN and cSN (cattle sialoadhesin) *N*-termini (excluding the 19-amino acid signal peptide) were modeled via homology modeling based on mSN (mouse sialoadhesin) template structures using bioinformatics tools. Subsequently, pSN and cSN homology structures were superposed onto the mSN protein structure to predict the binding sites of pSN. As a validation experiment, the SN *N*-terminus (including the wild-type and site-directed-mutant-types of pSN and cSN) was cloned and expressed as a SN-GFP chimera protein. The binding activity between SN and PRRSV was confirmed by WB (Western blotting), FAR-WB (far Western blotting), ELISA (enzyme-linked immunosorbent assay) and immunofluorescence assay. We found that the S107 amino acid residue in the pSN *N*-terminal played a crucial role in forming a special cavity, as well as a hydrogen bond for enhancing PRRSV binding during PRRSV infection. S107 may be glycosylated during PRRSV infection and may also be involved in forming the cavity for binding PRRSV along with other sites, including W2, Y44, S45, R97, R105, W106 and V109. Additionally, S107 might also be important for pSN binding with PRRSV. However, the function of these binding sites must be confirmed by further studies.

## 1. Introduction

Porcine reproductive and respiratory syndrome (PRRS) was first reported in North America in 1980 and has subsequently spread all over the world [1]. PRRS causes respiratory and reproductive disorders in pigs and leads to high mortality and enormous economic losses. PRRS is caused by the Porcine Reproductive and Respiratory Syndrome Virus (PRRSV), a positive sense RNA virus belonging to the order Nidovirales and family Arteriviridae [2]. PRRSV has two genotypes, European and American [3]. Pigs from any breed and at any age, whether male or female, can be infected at high viral loads by PRRSV. Pigs (wild or domesticated) are the natural hosts for PRRSV, and other animals cannot be infected by PRRSV [4].

Porcine alveolar macrophages (PAM) are one of the main target cells for receptor-mediated infection by PRRSV. Sialoadhesin (SN), heparan sulfate (HS) and CD163 have been demonstrated as PRRSV receptors on PAM [5]. PRRSV can also propagate in an African green monkey kidney cell line and cell lines derived from it, such as MA-104, Marc-145, Vero and CL-2621 [6].

CD163 is a specific hemoglobin in the Scavenger Receptor with Cysteine-Rich (SRCR) family that includes CD5, CD6, CD163, CD163b and WC1 [7]. CD163 is also necessary for full viral infection. The CD163 receptor plays a specific role in depolymerizing the virus capsid protein and releasing the viral genome after early adhesion [8].

HS glycoprotein, a cell membrane receptor, can also recognize and bind to several pathogens, such as Porcine Rabies Virus (PRV), classical Swine Fever Virus (SFV), Human Immunodeficiency Virus (HIV, the AIDS virus) and PPRSV [9]. Therefore, the HS receptor is not a PRRSV-specific receptor. HS can bind to the target cells in the early stages of PRRSV infection and is regarded as an adhesion factor.

SN is a sialic acid adhesion protein and macrophage-specific cell adhesion molecule and was originally named Sheep Red Blood Coagulation Receptor (SRBCR) [10]. SN has many domains, including 17 immunoglobulin-like domains, an *N*-terminal domain essential for sialic acid binding, a transmembrane domain and a short cytoplasmic tail [11]. SN is predominantly expressed in macrophage cells and can mediate their interaction with sialic acid [12]. Both SN and HS receptors are located on the PAM cell surface and play pivotal roles in PRRSV adhesion and internalization [13]. In non-permissive cells expressing both HS and SN receptors but not CD163, PRRSV can adhere and undergo endocytosis without the occurrence of complete viral shedding [14]. PRRSV infection can be inhibited by addition of the specific anti-SN monoclonal antibody 41D3 onto PAM cells, and HS cannot compensate for the SN because the SN receptor plays a more crucial role than the HS receptor in PRRSV infection [8]. Porcine Kidney cells (PK) are non-PRRSV-permissive cells because the SN protein is not expressed on the cell surface. However, PK cells expressing the *N*-terminal 150 amino acids (including the 19 amino acid signal peptide) of pSN can be infected by PRRSV. Expression of only the pSN *N*-terminus is sufficient to enable the process of virus adhesion [15]. Thus, the SN receptor is a key receptor for PRRSV infection, and the binding activity of the pSN *N*-terminal 150 amino acids is essential for the adhesion of sialic acid on the surface of PRRSV.

All PRRSV genotypes, including American and European PRRSV, interact with pSN via sialic acid on the surface of PRRSV [16]. PRRSV has many structural proteins, such as the GP1, GP2, GP3, GP4, GP5, M and N proteins. Among these proteins, only GP5 can bind to pSN on the envelope surface [17]. SN interaction and PRRSV infection can be reduced by removing sialic acid from the surface of the PRRSV GP5 protein [18]. Therefore, SN can interact with PRRSV directly through sialic acid on the surface of the PRRSV GP5 protein, and the sialic acid on the surface of the PRRSV GP5 protein is a key ligand of pSN [19].

**Figure 1.**
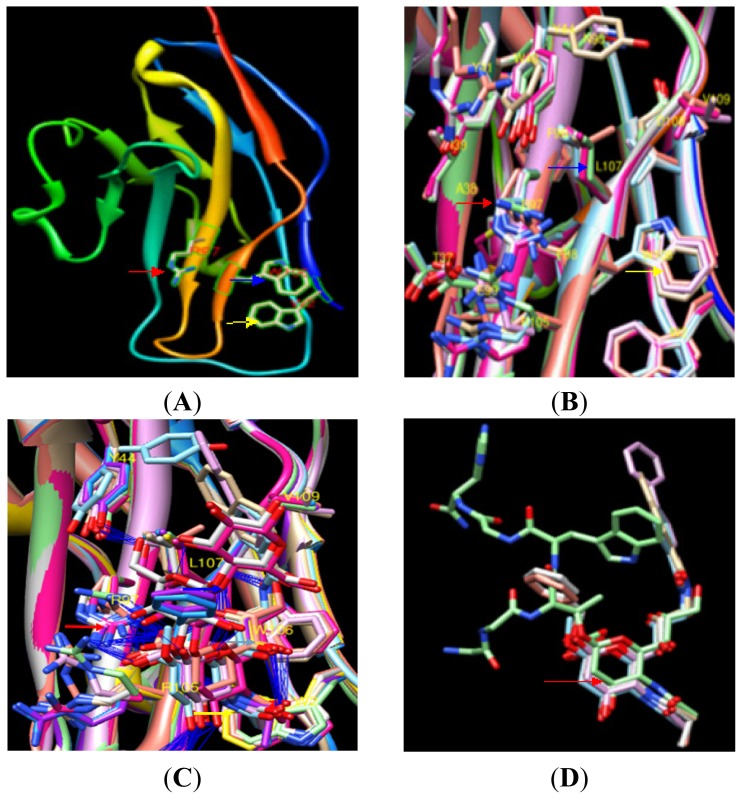
Skeleton model of the interaction of mSN with mouse sialic acid ligands. (**A**) Key amino acids in mSN have previously been reported to bind to sialic acid, including R97 (red arrow), W106 (blue arrow) and W2 (yellow arrow); (**B**) The backbones of six templates with their ligands deleted were superimposed using Chimera software (version 1.6.2, University of California, San Francisco, CA, USA, 2012); (**C**) The backbones of six templates along with their ligands were superimposed; Hydrogen bond interactions between ligands and mSN are denoted by a blue line. R97 (red arrow), W106 (blue arrow) and W2 (yellow arrow); (**D**) Ligand superimposition indicated that the position of the Neu-5Acα ligand core (red arrow) was conserved.

There are seven X-ray crystal diffraction structures of amino acids 1–119 (excluding the 19 amino acid signal peptide) of the mSN *N*-terminus in the Protein Data Bank (PDB), including PDB entries 1ODA, 1OD9, 1OD7, 1URL, 1QFO (a, b and c subunits), 1QFP and 2BVE (a and b subunits), for a total of 10 subunits in seven PDB structures (including PDB: 1QfO polymers with three similar trimers and PDB: 2BVE polymers with two similar dimer subunits) [20]. The other six mouse PDB structures with ligands (except for PDB: 1QFP without ligand) have crystal structures with their own mouse sialic acid-derived ligand. The W2, R97 and W106 sites (excluding the 19 amino acid signal peptide), along with other groups of amino acids in mSN, constitute a binding region for mouse sialic acid derivative ligand (Figure 1A) [21].

mSN can specifically recognize the Neu-5Acα-(2,3)Galβ(1,3)Gal-NAc sialic acid ligand structure. Neu-5Acα is located at the end of the sialic acid polysaccharide core [22]. If R97 is mutated to E97 in pSN, it prevents infection by PRRSV [23]. Thus, R97 could be the center of the sialic acid binding site in mSN and pSN, given the importance of this amino acid in the interaction with sialic acid.

Pigs (wild or domesticated) are the natural hosts for PRRSV, and other animals are not infected. pSN is the main receptor, and the pSN *N*-terminus is an important region for PRRSV infection [15]. However, the interaction between pSN and PRRSV and the exact locations of the pSN amino acids responsible for PRRSV binding are not well understood.

We previously predicted some of the other amino acid binding sites (with the exception of W2, R97 and W106) by applying bioinformatics methods to analyze the interaction of sialic acid with pSN or mSN, and we intended to identify and confirm the effect of the new binding sites. In this study, we constructed homologous structures of no-ligand pSN and cSN proteins based on mSN PDB templates, optimized the structures using molecular dynamics (MD) simulation and further analyzed the models. Furthermore, the predicted amino acids that could be essential for the interaction between PRRSV sialic acid and the SN receptor were mutated by site-directed mutagenesis, and their adhesion activities to PRRSV sialic acid were analyzed using WB, FAR-WB, ELISA and immunofluorescence assay compared with the wild-type SN.

This study aimed to investigate the interaction between pSN and PRRSV and the important regions and amino acid sites of the pSN *N*-terminal binding with PRRSV to understand the interaction between pSN and PRRSV and the differences in resistance or susceptibility to PRRSV infection observed in different species. This information may assist in the development of animal drugs and for breeding pigs with improved resistance to PRRSV infection.

## 2. Results

### 2.1. Bioinformatics Analyses

#### 2.1.1. Superimposition of mSN Templates with Ligand and Multiple Sequence Alignment of SN Protein

The similarity of DNA and amino acid sequences between pSN and mSN are very high (more than 85% and 65%, respectively [17]); thus, the binding region of pSN with the sialic acid on the surface of PRRSV may be similar between mSN and cSN. The homology model can achieve high accuracy when identity is more than the 65% required for protein modeling [24].

After superposing six PDB structures (nine subunits, excluding 1QFP no-ligand), the Root Mean Square Deviations (RMSD) in Å were very low among all subunits. Amino acids 1–119 (excluding the 19 amino acid signal peptides) were consistent with those from the PDB database (Table 1).

From the multiple sequence alignment of all the species (Figure S1), W2, Y44, R97, S103, R105, W106 and V109 were conserved. The sites at positions 103 and 107 were not conserved, and the sites at positions 3, 78 and 107, which had high predicted glycosylation scores, were also not conserved. The 107 site was V107 in cSN and L107 in mSN. pSN differed by harboring a hydrophilic amino acid at the S107 position.

#### 2.1.2. Identification of Amino Acid Binding Sites in mSN and pSN

The positions of six mSN PDBs (nine subunits, excluding 1QFP no-ligand) bound to mouse sialic acid derivatives as analyzed using Chimera software are summarized in Table 2. There were eight common amino acid binding sites among the six mouse PDB template structures (representing nine subunits), including W2, Y44, R97, S103, R105 W106, L107 and V109. All eight amino acids were near the R97 central binding site, and the distances between the PDB templates and the mouse sialic acid were within 6.5 Å.

W2, Y44, R97, S103, R105, L107 and V109 were interacting in the center of the mSN sialic acid binding region. In the mouse template structures, R97 and R105 could not be superposed very well because the conformation of two amino acid side chains was very flexible during the process of mSN binding to mouse sialic acid. Both R97 and R105 also provided hydrogen bonds by which mSN strongly bound with mouse sialic acid ligands.

However, the other amino acids were located according to their exact positions during mSN template superposition. The R97 at the center of the binding site was surrounded by a number of amino acids and sialic acids (Figure 1C). Although every sialic acid was different, all ligands had sialic acid Neu-5Acα as its core. Neu-5Acα was the sialic acid center, and its major part was highly conserved and stable (Figure 1D). In the PRRSV sialic acid binding process, PRRSV sialic acid may be fixed in this position and interact directly with R97. W2 and W106 were less flexible in that most large side chain structures of W2 and W106 did not change direction. During the process of PRRSV sialic acid binding with pSN, W2 and W106 might also be fixed and not moving (Figure 1B). W2 and W106 had no hydrogen bonds forming key sialic acid interactions but were restricted as to their sialic acid binding direction.

Therefore, according to mouse PDB results, these amino acids in pSN were similar to those in mouse and may be involved in binding sialic acid on the surface of PRRSV.

#### 2.1.3. Analysis of Signal Peptide and Glycosylation

The position of signal peptide cleavage was between 16–17 or 19–20 in pSN and cSN according to mSN PDB structures. But, the probable position was between amino acids 19–20 according to mSN templates. Glycosylation prediction showed that T1, T3, S6, T9, T78, T115 and T117 may be glycosylated in pSN (Figure S2). S107 appeared to have a low glycosylation score under normal conditions. According to the mouse PDB structure results, the bioinformatics analysis indicated that the S107 amino acid on the surface of pSN was near R97. We concluded that this amino acid interacting with the PRRSV should be glycosylated.

#### 2.1.4. Model Building by Superposition and Template Alignment

A resolution under 2.5Å for the structural homology modeling and full template were regarded as the basic standard in this study. The six mouse PDB subunits (excluding PDBs: 1QFO (c subunit), 1ODA, 1OD7 and 1QFP) that were under 2.5Å are marked by underlining in Table 1 and were used for our homology templates [25].

In accordance with the multiple sequence alignment results (Figure S3), nine models were constructed for cSN and pSN using MODELLER software (version 9v8, University of California, San Francisco, CA, USA, 2010). The cSN (Cattle.06) and pSN (Porcine.08) models were chosen as the candidate model structures based on the lowest Discrete Optimized Protein Energy (DOPE) scores from structures superposed to template. DOPE scores are shown in Table 3. From the multiple sequence alignment result, the 3, 78 and 107 amino acid sites were also not conserved in species homology modeling (Figure S1).

During glycosylation prediction, the T3 amino acid glycosylation score fell above the G-score. The multiple sequence alignment result for T78 located in the back of the pSN structure model showed that this residue differed between all species and had a high glycosylation score.

#### 2.1.5. Refinement of MD and Optimization of Homology Model

The best homology models, Cattle.06 for cSN and Porcine.08 for pSN, were analyzed by MD using Desmond software according to the default setting. Cattle.06 and Porcine.08 homology models were used to begin structural simulation in the MD process. The MD process ran for nearly 1440 ps. The model’s total energy, potential energy and RMSD in the protein backbone always changed with time during the MD process. After 1000 ps MD process, Cattle.06 and Porcine.08 became more stable and exhibited lowered energy compared with the beginning Cattle.06 and Porcine.08 structures. After 1000 ps, the backbone RMSD around Cattle.06 and Porcine.08 changed by less than 0.5 Å during the MD process (Figure S4). The final simulation conformation became stable.

Through MD process protein refinement and optimization, the stable structures for cSN and pSN were chosen for the next step of model quality assessment: superposition with each other and mutant analysis.

#### 2.1.6. Assessment of Model Quality

Ramachandran plot maps were used to check protein backbone and amino acid dihedral angle in the protein models. A higher Ramachandran plot map percentage corresponds to a better model quality [26]. First, the mSN PDB 1QFP (no-ligand) template structure was checked using the online PROBITY tool. The conformational region of the PDB 1QFP template before mSN binding with sialic acid was 88.03%.

In the Ramachandran plot map, pSN and cSN were approximately 89.74% and 87.39%. The Ramachandran plot map from six mouse PDB template structures with sialic acid ligand were as follows: mSN PDBs: 1QFO (a subunit) = 98.17%, 1QFO (b subunit) = 98.18%, 1OD9 = 94.87%, 2BVE (a subunit) = 91.45%, 2BVE (b subunit) = 93.16% and 1URL = 89.17%.

The six PDB mouse template structures binding with ligand could improve model quality because they exhibited higher Ramachandran plot map percentages than that of the 1QFP (no-ligand) PDB template structure (88.03%). This result showed that all of the mSN PDB templates with ligands were higher than the 1QFP (no-ligand) PDB. The pSN and cSN models without ligands had the lowest percentage Ramachandran plot map.

#### 2.1.7. Prediction of an Obvious Cavity in the pSN Surface Models for PRRSV Sialic Acid Binding

**Figure 2.**
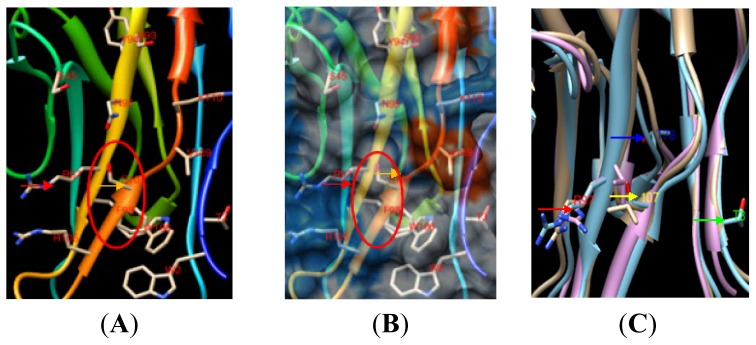
Skeleton model and surface model of pSN binding with PRRSV sialic acid. (**A**) In the pSN skeleton model, amino acids W2, Y44, S45, R105, W106, S107 (yellow arrow) and R97 (red arrow) formed an obvious cavity (red circle) to bind to PRRSV sialic acid; (**B**) In the pSN surface model, amino acids R97 (red arrow) and S107 (yellow arrow) formed a space for pSN to bind to PRRSV sialic acid (red circle); The blue surface indicates hydrophilic residues, and the red surface indicates hydrophobic residues; and (**C**) In skeleton model, superimposition of no-ligand pSN, mSN and cSN structures also showed that R97 (red arrow) and 107 (yellow arrow, including S107 in pSN, V107 in cSN and L107 in mSN) were important amino acids for binding, while the other amino acids including T3 (green arrow), T78 (blue arrow) were far away from R97, making it difficult for them to participate in the construction of the cavity.

In the cSN and pSN structures, no established ligand binding sites were observed, and mSN (PDB: 1QFP no-ligand) structures were superposed to identify differences using Chimera software. The RMSD between the three structure models was 0.874 Å in relation to pSN, which indicated that the three structures were highly similar (Figure 2A).

**Figure 3.**
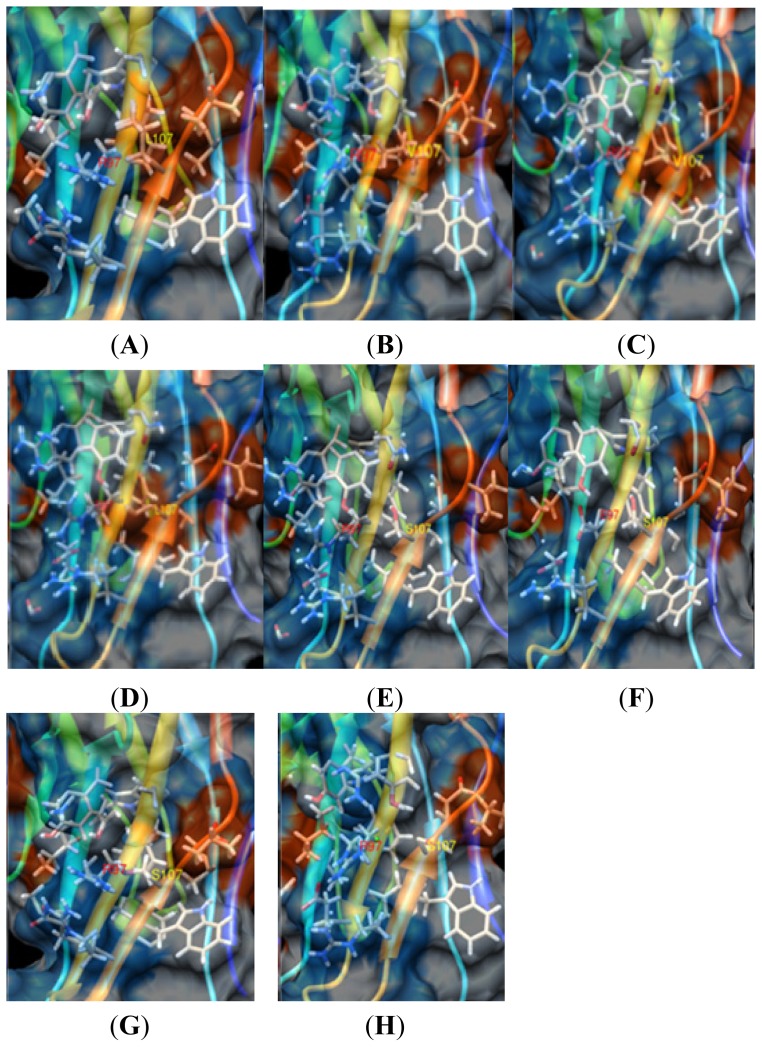
In the surface models of pSN, cSN and mSN binding to PRRSV, a large cavity and a hydrophilic site near the binding region were necessary for binding (blue, hydrophilic; red, hydrophobic; black, R97 and yellow, 107 (S107 in pSN, V107 in cSN and L107 in mSN)). (**A**) The cavity size of wild-type mSN 1QFP was 0 Å^3^, and with the L107 hydrophobic site, it may not bind to PRRSV sialic acid; (**B**) The cavity size of wild-type cSN was 84.5 Å^3^. With the L107 hydrophobic site, it could not bind to PRRSV sialic acid; (**C**) The cavity size of the S107V mutated pSN was 29.6 Å^3^. With the V107 hydrophobic site, it could not bind to PRRSV sialic acid; (**D**) The cavity size of S107L mutated pSN was 29.6 Å^3^. With the L107 hydrophobic site, it could not bind to PRRSV sialic acid; (**E**) The cavity size of wild-type pSN was 129.6 Å^3^ as calculated online by CAST. With its S107 hydrophilic site, pSN could bind well to PRRSV sialic acid; (**F**) The cavity size of R97E mutated pSN was 142 Å^3^, and it could not bind to the electronegative PRRSV sialic acid; (**G**) The cavity size of L107S mutated mSN was 133.1 Å^3^. With the S107 hydrophobic site, it may bind to PRRSV sialic acid; and (**H**) The cavity size of V107S mutated cSN was 77.2 Å^3^. With the S107 hydrophilic site, it could bind to PRRSV sialic acid;

However, in pSN, R97 and other amino acids formed an obvious cavity containing a hydrophilic pedestal (Figure 2B,C). The red circle represents the cavity’s position. W2, Y44, S103, R105, W106, S107 and V109 were positioned in different directions away from the cavity and formed a bigger erect space (Figure 2B,C), which likely provided accommodation for the sialic acid on PRRSV and may be beneficial to the PRRSV sialic acid interaction. In mSN, the corresponding region did not form a cavity in mSN (Figure 3A). Sites 3 and 78, which were not conserved between species in the SN multiple sequence alignment, were far away from the middle binding site, and T78 was in the back of pSN. Although the difference in T3 across the multiple species sequence alignment was also reflected in a high glycosylation score, it would be difficult for T3 and T78 to construct a cavity for PRRSV sialic acid binding.

Sites 97, 105 and 107 in pSN, cSN and mSN could not themselves be superposed very well in the superposing process (Figure 2A). These amino acids conformations were also different, which may change their direction when they are bound to their respective sialic acid.

The 107 amino acid in SN was different between pSN, cSN and mSN. A hydrophilic amino acid (S107) was observed in pSN, and its side chain was exposed and conveniently poised for interaction with PRRSV sialic acid (Figure 2A,B) based on the space surrounding S107. The hydrophobic amino acids L107 in mSN (Figure 3A) and V107 in cSN (Figure 3B), however, could not engage in polar bonds. Their direction was turned toward the inside of the space, and they were thus not readily suitable for adhesion to PRRSV sialic acid.

#### 2.1.8. Analysis of the Effect of pSN S107 on the Formation of the Binding Cavity

The S107 in pSN was near the R97 binding site, and therefore, replacements of amino acids at S107 and R97 in pSN could affect protein surface properties. The hydrophilic S107 in pSN was replaced by the hydrophobic V107 of cSN using Chimera software (version 1.6.2, University of California, San Francisco, CA, USA, 2012) (Figure 3C), while the L107 of mSN (Figure 3D) and other sites were unchanged. Hydrophobic V107 and L107 in pSN should not easily bind to the hydrophilic PRRSV sialic acid. There were not sufficient dedicated hydrogen bonds to allow PRRSV sialic acid binding. Another factor that could prevent PRRSV sialic acid binding upon mutation of R97 to E97 in pSN are protein side chains (Figure 3E,F). A previous study by Delputte and colleagues showed that in PK cells transfected with the pSN mutant pSN-R97E and infected by PRRSV, the pSN-R97E mutant reduced PRRSV infection relative to cells transfected with wild-type pSN [23]. The electric charge of sialic acid on the surface of PRRSV has been directly shown to be negative, similar to the also electronegative E97 residue but unlike the electropositive R97 [27]. Electronegative E97 could not bind with electronegative PRRSV sialic acid. Additionally, E97 electronegative amino acid hydrogen bonds were shorter than those formed by R97 in pSN. These conditions led to many disturbances to sialic acid binding, making PRRSV sialic acid binding to pSN-R97E difficult.

In mSN, the side chain residues are turned toward the inside of the protein and seal the cavity (Figure 3A,G) such that the cavity space becomes smaller. In mSN, the hydrophobic L107 residue was also not good at binding to PRRSV sialic acid, while a large cavity and necessary hydrophilic sites were very favorable for PRRSV sialic acid binding via hydrogen bonds in porcine wild-type pSN (Figure 3E). Mutating V107 to S107 in cSN with other amino acids unchanged was comparable to wild-type cSN (Figure 3B,H). In this case, the hydrophobic region was changed to a hydrophilic region and may lead to easier binding to PRRSV sialic acid. Mutation of L107 to S107 in mSN (Figure 3A,G) resulted in a bigger cavity, and the hydrophilic site may bind to PRRSV sialic acid.

Thus, S107 was very important in the formation of the cavity, which was a key condition for PRRSV sialic acid binding.

### 2.2. Experimental Validation

#### 2.2.1. SN-GFP Chimera Protein Expression in 293T Cell and Confirmation Using WB

To test the differential binding activity of SN with PRRSV, six chimeric SN-GPF proteins without M (trans-membrane domain) were cloned and expressed.

Six pEGFP-N1 vectors (two wild-types: cSN-GFP and pSN-GFP; four different mutants: pSN-S107V-GFP, pSN-T3V-GFP, pSN-T78V-GFP and cSN-V3T-V78T-V107S-GFP) were transfected into 293T cells to express the 150 (including the 19 amino acid signal peptide) *N*-terminal amino acids in SN along with GFP as a chimeric protein. The six SN-GFP chimeric proteins were expressed and secreted into cell medium. The proteins in the cell medium were collected and purified using protein ultra-filtration.

**Figure 4.**
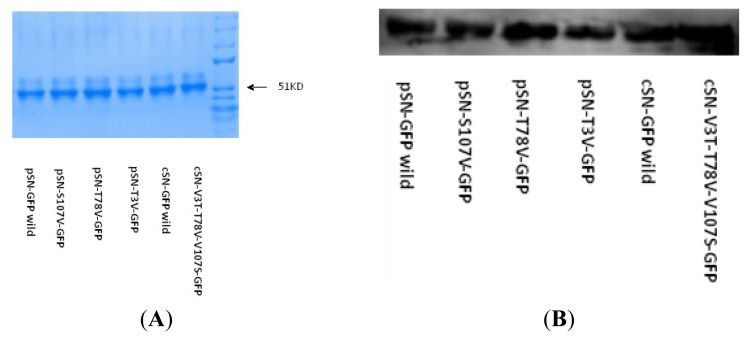
(**A**) The six different were SN-GFP proteins were expressed in 293T cells using the pEGFP-N1 vector and purified from cell medium by ultra-filtration such that the main component in the non-reducing PAGE were the SN-GFP chimeric proteins; and (**B**) The six different types of SN-GFP proteins identified by WB using a GFP-tagged antibody.

The purified proteins were separated using non-reducing PAGE and were located around 51 KD on the gel (Figure 4A). The six SN-GFP chimeric proteins were recognized using Western blot (WB) with GFP monoclonal antibody in a 1:2000 (Figure 4B).

#### 2.2.2. Analysis of the Binding Activity of SN-GFP Chimeric Proteins with PRRSV-GP5 Using FAR-WB

There are many structural proteins on the PRRSV membrane, including GP1, GP2, GP3, GP4 and GP5, but only the GP5 protein could bind to SN-GFP proteins, indicating that SN-GFP binding to PRRSV is in fact SN-GFP binding to PRRSV-GP5. Therefore, we used FAR-WB to analyze the binding activity of the six SN-GFP chimeric proteins with PRRSV-GP5.

**Figure 5.**
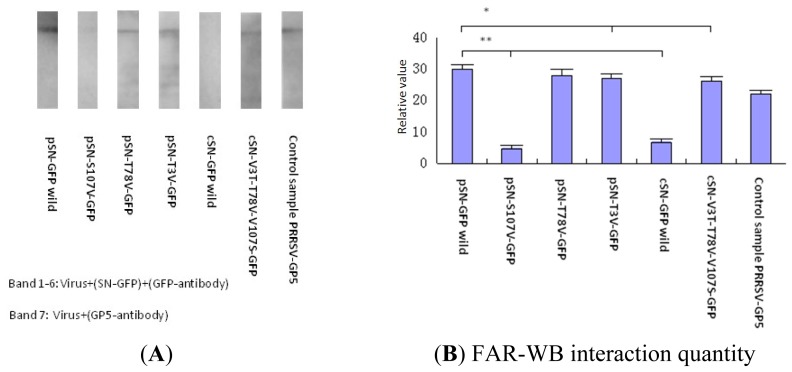
Binding activity analysis of SN-GFP proteins to PRRSV-GP5 using FAR-WB. (**A**) Binding activity of the six SN-GFP proteins with PRRSV-GP5 was tested using FAR-WB using a GFP-tagged antibody (bands 1–6). PRRSV-GP5 control samples were probed with anti-PRRSV-GP5 antibody (band 7); and (**B**) Binding activity of the six SN-GFP proteins with PRRSV-GP5 using FAR-WB. * significance; ** very significance.

Figure 5A shows that the five pSN-GFP chimeric proteins (wild-type, pSN-T78V-GFP, pSN-T3V-GFP, cSN-V3T-T78V-V107S-GFP and PRRSV-GP5) bound to PRRSV-GP5, whereas the pSN-S107V-GFP and wild-type cSN-GFP chimeric proteins did not bind.

In Figure 5B, wild-type pig SN (pSN-GFP) shows a much higher ability to bind PPRSV-GP5 compared with wild-type cattle SN (cSN-GFP) (** *p* < 0.01), which may explain why pigs are the natural hosts of PRRSV, while cattle is not permissive to PRRSV infection.

Compared with wild-type pig SN (pSN-GFP), the binding activity of the pSN-S107V-GFP and pSN-T3V-GFP mutants was significantly decreased (** *p* < 0.01 and * *p* < 0.05, respectively). The binding activity of the cSN-V3T-T78V-V107S-GFP mutant compared with pSN-GFP was significantly higher than that of wild-type cattle SN (cSN-GFP) (* *p* < 0.05).

Therefore, the pSN 107 site was very important in binding PRRSV.

#### 2.2.3. Analysis of PRRSV Binding to SN-GFP Chimeric Proteins Using ELISA

The concentrations of PRRSV and SN-GFP were tested using PRRSV-ELISA and GFP-ELISA kits, respectively, and equivalent amounts of PRRSV or SN-GFP were utilized in this experiment.

**Figure 6.**
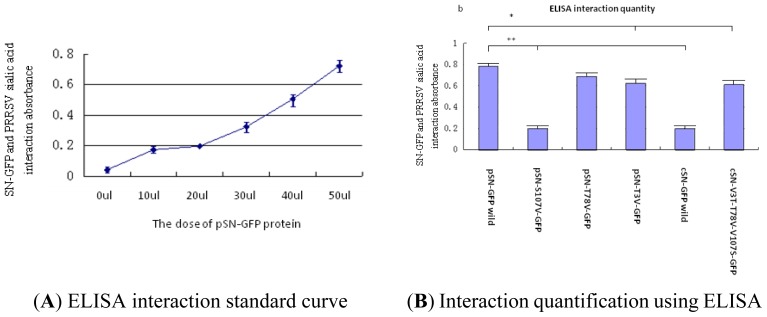
Binding activity of PRRSV to six different types of SN-GFP chimeric proteins were tested using ELISA. (**A**) Standard curve for the binding activity of different concentrations of wild-type SN-GFP with PRRSV; and (**B**) Binding activity of PRRSV to the different SN-GFP chimeric proteins. * significance; ** very significance.

ELISA was also used to test the PRRSV binding activity of the SN-GFP chimeric proteins. (Figure 6A) shows a standard curve for the SN-GFP chimeric protein interactions with PRRSV by ELISA analysis. The binding activity of wild-type pSN with PRRSV (30 ng/L) increased linearly with increased doses of SN-GFP protein when the pSN-GFP concentration was more than 20 μL.

The interactions of different types of SN-GFP proteins with PRRSV detected using ELISA (Figure 6B) were similar to the results obtained using FAR-WB (Figure 5B). Wild-type cSN-GFP and pSN-S107V-GPF did not bind to PRRSV sialic acid, whereas cSN-V3T-T78V-V107S-GFP, pSN-T3V-GFP and pSN-T78V-GFP obtained higher optical density values. Wild-type pSN-GFP generated the highest optical density value (Figure 6B).

Therefore, the ELISA results also hinted that the mutation of site 107 in pSN affected the binding activity of PRRSV and SN-GFP.

#### 2.2.4. Analysis of the Binding Activity of SN-GFP-M Chimeric Proteins with PRRSV Using Immunofluorescence

**Figure 7.**
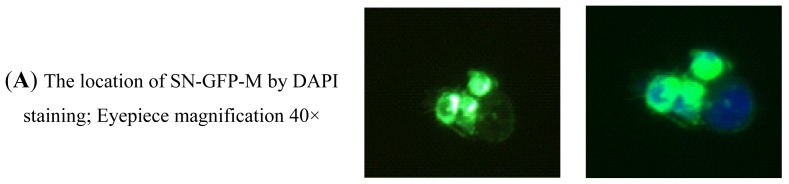

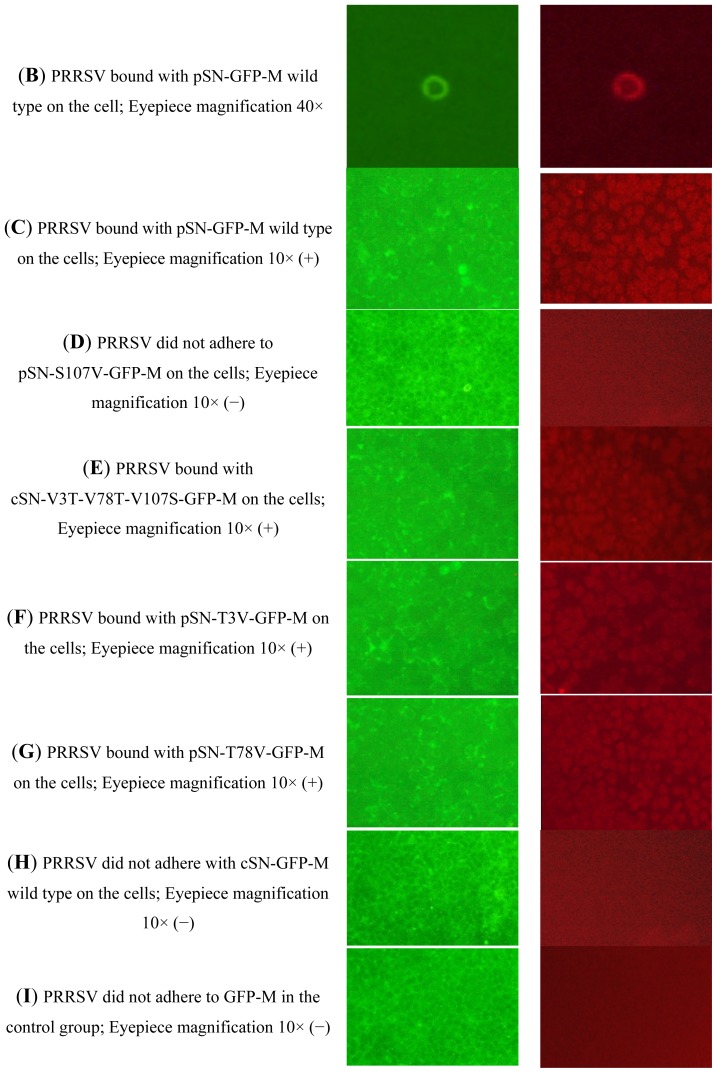
Analysis of the PRRSV binding activity of SN-GFP-M chimeric protein expressed in 293T cells using an immunofluorescence assay. (**A**) The SN-GFP-M chimera protein was located in the cytoplasm and on the membrane of 293T cells, as shown by DAPI staining (40× magnification); (**B**) Wild-type pSN-GFP-M chimera protein was expressed (left panel) and bound by PRRSV labeled with rhodamine red (right panel), 40× magnification; and (**C**–**I**) The left figure panels show the expression of SN-GFP-M in 293T cells, and the right figure panel indicates if binding of the different SN-GFP-M to PRRSV has occurred. “+” denotes binding of SN-GFP-M to PRRSV; and “−” denotes lack of binding between SN-GFP-M and PRRSV.

SN-GFP-M chimeric protein contains the 23 amino acids from the M trans-membrane region, the 150 *N*-terminal amino acids from SN (including the 19 amino acid signal peptide) and GFP, and was expressed in 293T cells. Some SN-GFP-M chimeric proteins were expressed both in the cytoplasm and on the membrane of 293T 8 hr after transfection (Figure 7A, 40× magnification).

Because the PRRSV was labeled with rhodamine red using an anti-PRRSV M protein polyclonal antibody, the cells appeared red when wild-type pSN-GFP-M bound to PRRSV (Figure 7B, 40× magnification; C, 10× magnification).

The cells expressing cSN-V3T-T78V-V107S-GFP-M, pSN-T3V-GFP-M and pSN-T78V-GFP-M also generated some red fluorescence because these SN-GFP-M chimeric proteins could bind with PRRSV (Figure 7E–G, 10× magnification).

However, red immunofluorescence was not observed in cells expressing cattle wild-type cSN-GFP-M, mutant pSN-S107V-GFP-M or the GFP-M control group. Those cells were not infected by PRRSV because the SN-GFP-M proteins could not bind to PRRSV (Figure 7D,H,I, 10× magnification).

Therefore, all of the experiments described above demonstrated S107 in pSN to be the key position for PRRSV binding to SN.

## 3. Discussion

PRRSV can only infect pigs [4], and PAM cells are the main host cells for PRRSV infection because there are abundant pSN receptor proteins on the surface of PAM cells [5]. PK cells are non-PRRSV-permissive but can bind the virus when pSN is expressed on the PK cell surface [28]. The process of pSN adhesion greatly affects the efficiency of PRRSV infection [29]. Some studies have shown that expression of SN in cells cannot lead to complete viral infection alone [14]. SN expression results in virus internalization into cells but not in uncoating, and thus, virus genomes are not released. pSN binding to PRRSV is a necessary step for further interaction of virus with CD163 [15]. Expression of only the pSN *N*-terminus is sufficient to enable virus binding with cells in a short time. Therefore, pSN is the key receptor, and the pSN *N*-terminus is an important region for PRRSV infection [15]. However, the interaction between pSN and PRRSV and the exact pSN amino acid sites responsible for binding to PRRSV must be further investigated.

The mSN PDB structure templates can bind to sialic acid ligands According to the mouse PDB, the template Ramachandran map value of mSN binding with sialic acid is above 90%, whereas the value of mSN without binding sialic acid is below 90%. In protein structures, higher template Ramachandran map values typically represent more reasonable dihedral angles and structures [26]. Sialic acid binding can improve mSN structure quality and lower protein structure maintenance energy. One possible reason for this is that interactions between oligosaccharide sialic acid ligands and SN hydrogen bonds may resolve some unreasonable dihedral angles within protein backbones. To properly bind to sialic acid ligands, certain inherent structural faults in protein dihedral angle can be corrected [30]. pSN also binds to PRRSV sialic acid, possibly in a manner similar to mSN binding to sialic acid. PRRSV binds to pSN through sialic acid on the surface of PRRSV [16]. Among the PRRSV structure proteins GP1, GP2, GP3, GP4, GP5, M and N proteins, the PRRSV GP5 protein on the envelope surface can bind to wild-type pSN [17]. PRRSV infection and SN interaction can also be reduced by removing sialic acid from the surface of the PRRSV GP5 protein. Therefore, SN can interact with PRRSV directly through sialic acid on the surface of the PRRSV GP5 protein [18].

Mouse PDB templates were further analyzed to predict the binding sites of mSN with sialic acid eight common binding sites of amino acids (W2, Y44, R97, S103, R105, R106, L107 and V109) from six mouse PDB structures have been discovered. Amino acids R105, S103, R97 and Y44 interact with sialic acid through hydrogen bond formation, and hydrogen bond formation and protein structure maintenance energy are preserved. The key flexible amino acids forming hydrogen bonds can determine binding position and energy [31]. Our results indicated that W2, Y44, R97, S103, R105, R106, L107 and V109 may play important roles in mSN binding to sialic acid that amino acids R2, R97 and R106 were very important for interaction [22]. During SN structure superposition, mSN structure superposition has shown that W2 and W106 have a conserved direction and position in the mouse sialic acid core. These two amino acids also limit the direction of sialic acid binding, while the direction of R97 always changes when engaging in active hydrogen bond interactions [22] with flexible amino acids that can be used for hydrogen bond formation [31]. mSN mutagenesis experiments have further demonstrated that W2, R97 and W106 amino acids are very important in the interaction of mSN with sialic acid [22].

To predict the binding region and binding sites of pSN with PRRSV, we established homologous structures of pSN and cSN *N*-terminal, optimized these structures and analyzed the models based on the mSN PDB templates and the high degree of similarity between the pSN, cSN and mSN *N*-terminal protein sequences (more than 65% similarity) [15,32]. We found that R97 in pSN was also a flexible amino acid that could hydrogen bonds to the sialic acid Neu-5Acα core, implying that R97 in pSN may also be an important amino acid in the interaction with PRRSV sialic acid.

Some studies have demonstrated that the pSN R97E mutant (from R97 to E97) exhibits no binding to PRRSV sialic acid, significantly lowering PRRSV infection [23] and leading to deletion of hydrogen bonds due to the shorter side chain of E97 (compared with that of R97). During binding, PRRSV sialic acid is electronegative and cannot bind to an also electronegative E97 amino acid [21]. Additionally, R105, another flexible amino acid that is similar to R97, may also be important.

After CAST (online software: http://sts-fw.bioengr.uic.edu/castp/calculation.php) analysis, we found an obvious cavity in pSN (129.6 cubic Å in volume), whereas no cavity was observed in mSN or cSN. The amino acids W2, Y44, S103, R105, W106, S107 and V109 around R97 were necessary for cavity formation, and S107 in pSN was a hydrophilic amino acid forming one hydrogen bond supporting specific recognition and binding of sialic acid on the surface of the PRRSV GP5 protein. The hydrophobic amino acid L107 in mSN and the V107 amino acid in cSN could not supply hydrogen bonds. This observation hinted at one of the possible reasons why pigs are naturally infected by PRRSV. A large cavity means that the ligand-receptor interaction surface area is bigger than with no cavity [33], and a large cavity and enlarged binding surface also allow specific hydrogen bond interactions because the energy of sialic acid hydrophilic ligand binding is correlated with the number of hydrogen bonds [31].

To confirm that the S107 residue in pSN plays an important role in pSN binding of PRRSV, we performed validation experiments SN-GFP and SN-GFP-M chimeric proteins including wild-type pSN, point-mutated pSN, wild-type cSN and point-mutated cSN were expressed in 293T cells. 293T cells are highly adapted to SV40/CMV elements and are also suitable for chimeric protein expression in which proteins are secreted into the cell medium and easily extracted [34,35]. In our study, the main SN-GFP protein was confirmed by WB and GFP-ELISA.

The binding activities between SN-GFP and PRRSV-GP5, SN-GFP and PRRSV and SN-GFP-M and PRRSV were detected by FAR-WB, ELISA and immunofluorescence assays, respectively. These three experiments demonstrated that the binding activity of the mutant pSN-S107V-GFP chimeric protein where pSN S107 is mutated to cSN V107 was much lower than that of wild-type pSN-GFP.

The bioinformatics analyses in this study showed that the S107 in pSN was a hydrophilic amino acid, whereas V107 in cSN and L107 in mSN were hydrophobic amino acids. pSN-S107V and cSN-V107S mutants changed the hydrophobic amino acid or hydrophilic amino acid that may alter cavity size and hydrogen bond interaction with sialic acid. Hydrophilic amino acids, hydrogen bond interactions and cavity size were very important in the interaction between pSN and PRRSV sialic acid.

Thus, all of the experiments in this study revealed that that the S107 amino acid site residues in the pSN *N*-terminus may play a crucial role in forming a specialized cavity, as well as hydrogen bonds for enhancing PRRSV sialic acid binding during PRRSV infection.

For the other amino acids in this study, the effects of T3 and T78 binding with PRRSV were not as important as S107, likely because they were farther away from the binding center and cavity. pSN sites 3 and 78 did not play an important role in PRRSV infection despite being hydrophilic amino acids (similar to S107) with a predicted high glycosylation score.

## 4. Materials and Methods

### 4.1. Ethics Statement

All animal experiments were performed according to International Standards and Guidelines. Animal blood was collected from the Huazhong Agricultural Farm.

### 4.2. Bioinformatics Analysis

#### 4.2.1. Methods and Software

Homology models were generated using MODELLER (http://salilab.org/modeller/), and graphical displays were generated with Chimera software (http://www.cgl.ucsf.edu/chimera/) [24]. After homology models were built, Molecular Dynamic (MD) simulations for homology models using Desmond software were optimized and refined (http://www.deshawresearch.com/resources_desmond.html) [36]. Hydrophobic surface and mutations were analyzed with Chimera software [37]. SignalP 3.0/4.1 and NetOGlyc 3.1 servers were used (http://www.cbs.dtu.dk/services/SignalP/) for signal peptide prediction and glycosylation prediction (http://www.cbs.dtu.dk/services/NetOGlyc/) of SN, respectively [38,39]. An on-line PROBITY Ramachandran Map for protein backbone dihedral angles (http://molprobity.biochem.duke.edu/index.php) was used to check model quality, and CASTP (http://sts-fw.bioengr.uic.edu/castp/calculation.php) was used to calculate the surface cavity size of the SN protein [26,40].

#### 4.2.2. Analysis of the Interaction of mSN with Sialic Acid

Hydrogen bond interactions between amino acids and different sialic acid-derived ligands were analyzed using Chimera. The backbones of nine subunit template structures of six PDBs (excluding PDB: 1QFP without ligand) were superposed. Amino acid interactions within 6.5 Å of the R97 central binding site in mSN are shown (Figure 1B,C). The resolution of all PDB models was under 3.0 Å. Based on the properties of the mSN PDBs, we found conformational changes in flexible amino acids upon mSN binding to different sialic acid derivatives that may play important roles in sialic acid binding. Interactions of the pSN amino acids with sialic acid on the surface of PRRSV were then estimated.

#### 4.2.3. Modeling of pSN and cSN Structures without Ligand

Because the resolution of some mSN template structures was above 2.5Å, we used only the six mSN subunits with resolutions under 2.5Å (including PDBs:2BVE (a and b subunits), 1QFO (a and b subunits), 1URL and 1OD9 and excluding PDBs: 1ODA, 1OD7, 1QFO (c subunit) and 1QFP (no-ligand)) as templates to construct the no-ligand homology structures of the *N*-terminal 1–119 amino acids of the pSN and cSN proteins (excluding the 19 amino acid signal peptides). All homology models included the SN *N*-terminal 1–119 amino acids (excluding the 19 amino acid signal peptides) according to mouse templates. Homology models of pSN and cSN were produced nine at a time by the MODELER software. The best model was chosen based on the lowest Discrete Optimized Protein Energy (DOPE) score out of each set of nine models. We also checked the quality of the models by aligning them with the 1QFP PDB template (no-ligand) and by using PROBITY tool’s online Ramachandran Map [26]. Multiple sequence alignment was performed using Clustal X for homology models and for analysis of protein species alignment [41]. Sequence identities higher than 65% ensured homology model reliability [24].

The best cSN and pSN models from the MODELER homology model were treated with Desmond MD simulation software. The best model structures were optimized by applying the MD process using default settings [36].

#### 4.2.4. Analysis of the SN Protein Hydrophobic Surface and Prediction of Amino Acid Binding with Sialic Acid on PRRSV

The final MD simulation structures for pSN and cSN were aligned and superposed with the mouse PDB 1QFP (no-ligand) template (Figure 2A) to identify their structural differences and differences in hydrophobic surface using Chimera software. Next, we mutated S107 to V107 and R97 to E97 in pSN, leaving other amino acids unchanged. We also mutated V107 in mSN and L107 in cSN to S107. The criterion for amino acid mutation was having the lowest rotamer score in Chimera. CASTP was used to calculate the size of the special cavity near R97, and default 1.4 Å solvent probes were used in all of the CASTP calculation processes.

### 4.3. Experimental Methods

#### 4.3.1. PRRSV and TCID50 Assay

The WuHan strain of PRRSV is a mutated genotype belonging to the American genotype virus and is more prevalent and more serious than the European and American genotypes in China. Wuhan PRRSV was kindly provided by the College of Veterinary Medicine, Huazhong Agricultural University. A TCID50 of 10^4^ was quantified in MARC-145 cells after five passages. PRRSV was prepared for the TCID50 assay using the 10-fold dilution method in the MARC-145 cell line [42].

#### 4.3.2. RNA Extraction, cDNA Synthesis, Mutation of pSN and cSN and Construction of Expression Vector

The amino acid sites T3, T78 and S107 in pSN were likely important according to our software-based predictions. To test the roles of these amino acids in the binding activity of PRRSV with SN and with chimeric SN GFP-tagged proteins (SN-GPF without the SN M trans-membrane domain and SN-GPF-M with the SN M trans-membrane domain), site-directed mutants of pSN and cSN were cloned and expressed, along with wild-type versions of the proteins.

Total RNA was extracted from porcine and cattle whole blood using TRIzol reagent (Invitrogen, San Francisco, CA, USA) according to the manufacturer’s protocol, as described by Xu *et al*. [43]. One microgram of total RNA was reverse-transcribed using the First Strand cDNA Synthesis Kit following the manufacturer’s protocol (Fermentas, Toronto, ON, Canada) [44]. Primers in Table 4 were designed using PerlPrimer to amplify five (as shown in Table 4) wild-type and mutated cDNA fragments corresponding to 150 amino acids of the SN *N*-terminus, as well as the cDNA for the SN trans-membrane M domain from porcine and cattle. The PCR amplification program for all cDNAs consisted of a 10 min enzyme activation step at 95 °C, followed by 35–40 cycles of a 30 s denaturing step at 98 °C and a 30–45 s annealing/extension step at 53–56 °C, with a final extension step of 10 min at 72 °C. High-fidelity polymerase enzyme (Cw Biotech, Beijing, China) was used in all reactions. The three site-directed mutations in cSN (V3T, V78T and V107S) were synthesized by the Sangon Biological Engineering Technology Company (Shanghai, China).

Thirteen expression vectors were constructed using the pEGFP-N1 vector, including six vectors without the M trans-membrane domain of SN (two wild-types: cSN-GFP and pSN-GFP; four different mutants: pSN-S107V-GFP, pSN-T3V-GFP, pSN-T78V-GFP and cSN-V3T-V78T-V107S-GFP) for expression of the SN-GPF chimeric proteins (containing 150 amino acids of the SN *N*-terminus and GFP) and seven vectors with the M trans-membrane domain of SN (pSN-S107V-GFP-M, pSN-T3V-GFP-M, pSN-T78V-GFP-M, cSN-V3T-V78T-V107S-GFP-M, pSN-GFP-M, cSN-GFP-M and GFP-M control) for expression of the SN-GPF-M chimeric proteins. The SN *N*-terminus was inserted into the Bgl II and Age I enzyme Multiple Cloning Site (MCS) ahead of the vector’s Green Fluorescence Protein (GFP) sequence. The SN trans-membrane M domain was inserted using BsrG I and Xba I enzyme sites. All SN proteins were expressed as SN-GFP chimera proteins or as SN-GFP-M chimera proteins. All SN-GFP chimera proteins were secreted into the cell medium, and all SN-GFP-M chimera proteins were expressed in the cell membrane.

For the construction of mutation vectors, the Easy Mutagenesis System was used according to manufacturer’s instructions (TransGen Biotech, Beijing, China) [45]. Following amplification and ligation, different SN target fragments were fused into pEGFP-N1 vectors.

#### 4.3.3. Ultra-Filtration (UF) for Collection and Purification of SN-GFP Chimera Proteins from Cell Medium

SN-GFP chimera proteins from cell medium were collected using 10 and 100 KD ultra-filtration membranes (Millipore, Boston, MA, USA) [19]. The theoretical molecular weight of wild-type SN-GFP was 43 KD (26 KD GFP + 17 KD pSN *N*-terminus) according to theoretical calculations based on the number of amino acids.

Cells were removed from supernatant medium by centrifugation at 500 rpm for 5 min. Spent medium was collected and filtered through 0.22-μm filters. All operations were performed at 4 °C, and protein was gently extracted with care not to damage the cell membrane. Filtered supernatant medium was then centrifuged at 2000 rpm for 2 min. Supernatant medium was filtered through 100 KD ultra-filtration membranes and blocked by 10 KD ultra-filtration membranes. Protein was collected and preserved in −80 °C for further analysis.

#### 4.3.4. Non-Reducing PAGE, WB and FAR-WB Detection of PRRSV-GP5 Binding Activity and SN-GFP Chimera Proteins

Non-reducing PAGE was used to separate the extracted proteins from cell medium, and WB was used to confirm SN-GFP chimera proteins on non-reducing PAGE. Proteins (10 μL) were mixed with 10 μL of loading buffer (Cw Biotech, Beijing, China) and separated under non-reducing conditions using NuPAGE 8%–10% Bis-Tris Gels. Protein was electro-transferred to 0.45-μm PVDF membranes (Cw Biotech, Beijing, China). Membranes were blocked with blocking buffer containing 2% BSA (QIAGEN, Düsseldorf, Nordrhein-Westfalen, Germany) for 1 h and incubated overnight at 4 °C with 1:2000 mouse anti-GFP monoclonal antibody (Proteintech, Chicago, IL, USA), followed by washing with TBST (containing 0.05% Tween-20) buffer. Peroxidase-conjugated goat anti-mouse IgG antibody was then added for 1 h at room temperature.

FAR-WB was used to detect the interaction of SN-GFP with PRRSV sialic acid. PRRSV was mixed with a protein loading buffer that lysed PRRSV and released PRRSV proteins, and PRRSV proteins were separated by non-reducing PAGE. PRRSV protein samples were electro-transferred onto 0.45-μm PVDF membranes (Cw Biotech, Beijing, China). Membranes were blocked with blocking buffer containing 2% BSA (QIAGEN, Düsseldorf, Nordrhein-Westfalen, Germany) and incubated with the six different SN-GFP chimera proteins overnight at 4 °C. The control group was a PRRSV-GP5 protein sample that could be directly visualized by treatment with a 1:1000 dilution of mouse anti-PRRSV GP5 protein (Whboster, Wuhan, Hubei, China) following a traditional WB method. In FAR-WB, the primary antibody, a mouse anti-EGFP monoclonal antibody (Proteintech, Chicago, IL, USA) was incubated on the membrane for 2 h. The membrane was then washed with TBST (containing 0.05% Tween-20). The secondary antibody, 1:3000 Horseradish Peroxidase (HRP)-conjugated goat anti-mouse IgG, was incubated with the membrane for 1 h at room temperature. In the control group, after PRRSV-GP5 binding with mouse anti-PRRSV GP5 protein monoclonal antibody, 1:3000 goat anti-mouse HRP-IgG was added and incubated for 1 hr at room temperature. The bands were visualized using the ECL substrate (QIAGEN, Düsseldorf, Nordrhein-Westfalen, Germany) detection system (Image Quant LAS 4000 mini, GE Healthcare, London, UK) and software [46].

All bands on the membranes were collected and scanned, and the relative contents of the band compared with the membrane background were converted into numeric values using Image J software (http://rsbweb.nih.gov/ij/) [47]. The differences between the values of the bands compared with those of the wild-type pSN-GFP bands were analyzed with SPSS 18 (Statistical Product and Service Solutions, Version 18.0, IBM, Chicago, IL, USA, 2010).

#### 4.3.5. ELISA for Detection of Interactions between PRRSV and SN-GFP Chimeric Proteins

PRRSV was concentrated using 300 KD ultra-filtration membranes. To use the same amount of PRRSV and SN-GFP in the experiment detecting interaction of PRRSV and SN-GFP chimera proteins, the concentration of PRRSV and GFP-tagged (SN-GFP) protein were measured using PRRSV and GFP ELISA Kits according to the supplied protocol (Cell BioLabs, San Diego, CA, USA).

Each double antibody sandwich ELISA kit contained its own anti-GFP or anti-PRRSV first antibodies. In both commercial double antibody sandwich ELISA kits, some anti-PRRSV or anti-GFP antibody came fixed onto the assay plates [48].

The ELISA kit was used to test the interaction of PRRSV and SN-GFP. PRRSV (50 μL) was added to each well, in which anti-PRRSV antibody had been fixed on the ELISA plate and incubated at 37 °C for 30 min. The plate was then washed five times using 0.01 mol/L PBS wash buffer, and 10–50 μL of SN-GFP protein was then added. The ELISA plate was then blocked using 2% BSA for approximately 1 h and washed five times using wash buffer. Anti-GFP antibody (50 μL) (not anti-PRRSV) from the GFP ELISA kit was added to the PRRSV ELISA plate and incubated at 37 °C for approximately 30 min. After washing, 50 μL of the HRP-conjugated secondary antibody from the GFP ELISA kit was added to the PRRSV ELISA plate and incubated at 37 °C for approximately 30 min.

Finally, the plate was washed five times with 0.01 mol/L PBS washing buffer and mixed with 50 μL of TMB substrate at 37 °C for 15 min, after which 50 μL of stop buffer was added. The depth of color showed the strength of interaction between PRRSV sialic acid and SN-GFP.

#### 4.3.6. Immunofluorescence Assay to Test the Interaction between PRRSV and SN-GFP-M Chimeric Proteins

293T cells were seeded in 24-well culture plates and cultured until reaching approximately 85% confluence. Cells were then washed with DMEM (without antibiotic and antifungal agents) three times. Cells were transfected by SN-GFP-M plasmids using a lipofectamine method following the manufacturer’s protocol (Roche, Basel, Switzerland) [49].

Under normal conditions, cells were cultured in 10% FBS, and GFP fluorescence was checked with a fluorescence microscope (NIKON fluorescence microscope, Nikon, Tokyo, Japan) after 8 h. The expression of the six SN-GFP-M as well as the GFP-M were checked at 488 nm absorbance.

Immunofluorescence assay was also used to assess the interaction of PRRSV sialic acid and SN-GFP-M. Each well was washed for three times with PBS 0.01 mol/L. Transfected cells fixed with 4% polysorbate were washed three times with 0.01 mol/L PBS and blocked with 30 μL of 2% BSA in PBS (0.01 mol/L) at 37 °C for 1 h. Virus was added to each well (30 μL total volume) and incubated at 37 °C for 1 h. The anti-PRRSV M antibody was a polyclonal antibody directly labeled with rhodamine red (Bioss, Beijing, China). Antibody was added to each well in a 1:150 dilution in a total volume of 30 μL, incubated at 37 °C for 1 h and then washed with PBS (0.01 mol/L) three times. Finally, 0.1% DAPI was added for staining of cell nuclei.

SN-GFP-M and DAPI expression were analyzed using 488 nm and 358 nm wavelengths using a fluorescence microscope. Immunofluorescence was performed to test the signals of GFP and PRRSV-rhodamine red at 488 and 552 nm, respectively. In 293T cells, all ligands and receptor interaction experiments were completed within 6 h [16].

## 5. Conclusions

In this study, we performed predictions of binding sites of pSN with sialic acid on the surface of PRRSV using bioinformatics analyses and further detected and analyzed the binding activities of PRRSV and pSN in wild-type and several site-directed pSN and cSN mutants using WB, FAR-WB, ELISA and immunofluorescence cell assay.

We found that residue S107 in pSN was crucial for PRRSV infection and formed a cavity with other amino acids to enable pSN binding to PRRSV sialic acid. In addition to the S107 site, other pSN amino acids, including W2, Y44, S103, R97, R105, W106 and V109, may also be involved in PRRSV infection, a finding that requires further experimental confirmation. These results may help to understand the interaction between pSN and PRRSV and the differences in resistance or susceptibility of different species to PRRSV infection, as well as provide information for the development of animal drugs and pig breeding strategies to improve resistance to PRRSV infection.

## Supplementary Information



## Figures and Tables

**Table 1.** Template protein backbone RMSD between mSN subunits (excluding 1QFP no-ligand).

RMSD	PDB:ID	1OD7	*1OD9*	1ODA	*1QFOa*	*1QFOb*	*1QFOc*	*1URL*	*2BVEa*	*2BVEb*
1OD7										
***1OD9***		0.447 Å								
1ODA		0.434 Å	0.439 Å							
***1QFOa***		0.468 Å	0.421 Å	0.454 Å						
***1QFOb***		0.591 Å	0.585 Å	0.476 Å	0.418 Å					
***1QFOc***		0.407 Å	0.331 Å	0.470 Å	0.400 Å	0.394 Å				
***1URL***		0.551 Å	0.541 Å	0.571 Å	0.518 Å	0.570 Å	0.491 Å			
***2BVEa***		0.509 Å	0.455 Å	0.561 Å	0.417 Å	0.437 Å	0.447 Å	0.562 Å		
***2BVEb***		0.438 Å	0.441 Å	0.449 Å	0.339 Å	0.347 Å	0.356 Å	0.564 Å	0.375 Å	

Underlining and italic denotes a homology template under 2.5 Å.

**Table 2.** Interactions of mSN amino acids with sialic acid.

PDB:ID	Subunit	Amino acids binding to sialic acid									
1ODA		W2	N95	***R97***	***S103***	***R105***	W106	L107	D108	V109	
1OD9		W2	***Y44***	***R97***	***S103***	***R105***	W106	L107	V109		
1OD7		W2	***Y44***	S45	***R97***	***S103***	***R105***	W106	L107	D108	V109
1URL		W2	***R97***	***R105***	R106	L107	V109				
2BVE	A	W2	***R97***	***S103***	***R105***	R106	L107	V109			
	B	W2	***R97***	***S103***	N104	***R105***	W106	L107	V109		
1QFO	A	W2	***Y44***	***R97***	***S103***	***R105***	W106	L107	V109		
	B	W2	***Y44***	***R97***	***S103***	***R105***	W106	L107	V109		
	C	W2	***R97***	***S103***	***R105***	R106	L107	V109			
1QFP											

Underlining and italic indicates that amino acids form hydrogen bonds. 1QFP no-ligand.

**Table 3.** DOPE scores of cattle and porcine models.

Model name	DOPE score
Cattle.01	−12,062
Cattle.02	−12,184
Cattle.03	−12,156
Cattle.04	−12,079
Cattle.05	−12,142
***Cattle.06***	−***12,238***
Cattle.07	−12,052
Cattle.08	−12,217
Cattle.09	−12,151
Porcine.01	−12,425
Porcine.02	−12,506
Porcine.03	−12,270
Porcine.04	−12,503
Porcine.05	−12,543
Porcine.06	−12,570
Porcine.07	−12,509
***Porcine.08***	−***12,599***
Porcine.09	−12,486

Underlining and italic denotes chosen candidate models: Cattle.06 for cSN and Porcine.08 for pSN.

**Table 4.** Primers used to construct six SN *N*-terminal and trans-membrane domains.

SN	5′ terminal primer	3′ terminal primer	Annealing temperature
pSN T3G	GGCCTGGCCTCGTGG ggc GTTTCCAGCCCCGAGA	TCTCGGGGCTGGAAAC gcc CCACGAGGCCAGGCC	54.0 °C
pSN T78V	CAGGTTGAACAGAGG gtg TGCAGCCTGCTGCTG	CAGCAGCAGGCTGCA cac CCTCTGTTCAACCTG	54.0 °C
pSN S107V	GAGGGCAACCGCTGG gta GATGTCAAAGGCACAG	CTGTGCCTTTGACATC tac CCAGCGGTTGCCCTC	54.5 °C
pSN Wild-type	GTAGATCTTC ATGGACTTCCTGCTCCTGCTCCTC	CAACCGGTAA CAAGGCAATGGTGGGCACGCTGG	55.0 °C
cSN Wild-type	GTAGATCTTC ATGGACTTCCTGCTCCAGCTCCTC	CAACCGGTAA AGAGGCAACCGTGGGCATGATGAG	56.0 °C
SN membrane domain BsrG I/Xba I	CTTGTACAAGCTTCTCTGGTTCCTGG	GCTCTAGACTACCAGACCCCCAGGCC	53.0 °C

Lower-case letters denote mutations.

## Abbreviations

PRRS: Porcine Reproductive and Respiratory Syndrome
SN: Sialoadhesin
pSN: porcine Sialoadhesin
cSN: cattle Sialoadhesin
mSN: mouse Sialoadhesin
SRBCR: Sheep Red Blood Coagulation Receptor
IG: Immuno Globulin
PDB: Protein Data Bank
UF: Ultra-filtration
RMSD: Root Mean Square Deviation
PS: Picosecond
SRCR: Scavenger-Receptor with Cysteine-rich family
MD: Molecular Dynamics
PRV: Porcine Rota Virus
SFV: Swine Fever Virus
HIV: Human Acquired Immune Deficiency Syndrome Virus
HS: Heparan Sulfate
DOPE score: Discrete Optimized Protein Energy score
Å: Angstrom
PAM: Porcine Alveolar Macrophages
PBMC: Porcine Blood Mononuclear Cells
PK: Porcine Kidney cells
FAR-WB: Far Western Blotting
PAGE: Polyacrylamide Gel Electrophoresis
MCS: Multiple Cloning Site
