# Supplementary material for: Analysis of the Binding Sites of Porcine Sialoadhesin Receptor with PRRSV
Source: Int J Mol Sci. 2013 Dec 9;14(12):23955–79. doi: 10.3390/ijms141223955 (PMC3876088; doi:10.3390/ijms141223955)
Supplement: Supplementary file 1 [file ijms-14-23955-s001.pdf]

Supplementary Information

Figure S1. Multiple alignment of 1-180 amino acids sequences at N-end protein of pSN, cSN and mSN. The three species SN had 19 amino acids signal peptide. Red arrow indicated signal peptide cutting site. Key amino acids binding with PRRSV included R97 (R116 with 19 amino acids signal peptide, black arrow), S107 (S126 = 107 + 19 amino acids signal peptide, black arrow) and T3 (3 + 19 = 21, yellow arrow) with high glycosylation score T78 (78 + 19 = 97, yellow arrow). The identification of amino acids sequence among three species in SN was > 65%. The sequence homology > 65% ensured homology model accuracy.

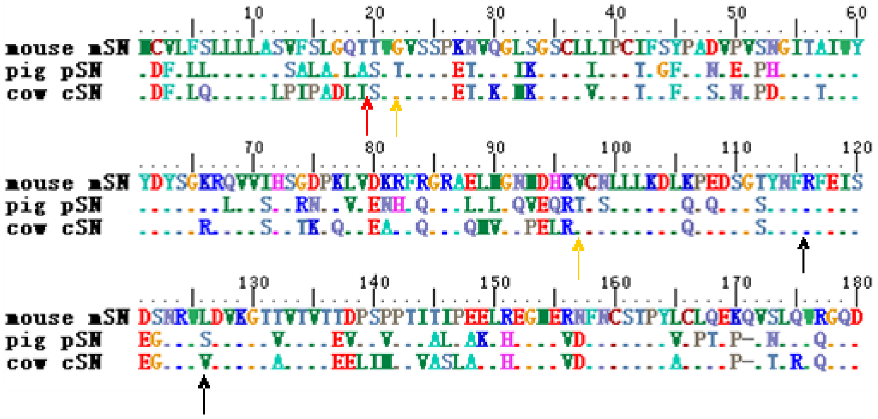

Figure S2. (**A**) Signal peptide prediction. The most probable position was between 19-20 amino acids; (**B**) Glycosylation prediction. Glycosylation prediction showed that T1, T3, T9, T78, T115, T117 and S6 were glycosylated in pSN with high scores.

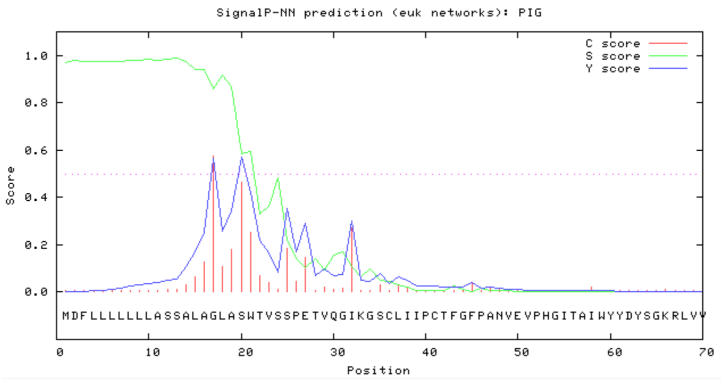

(**A**)

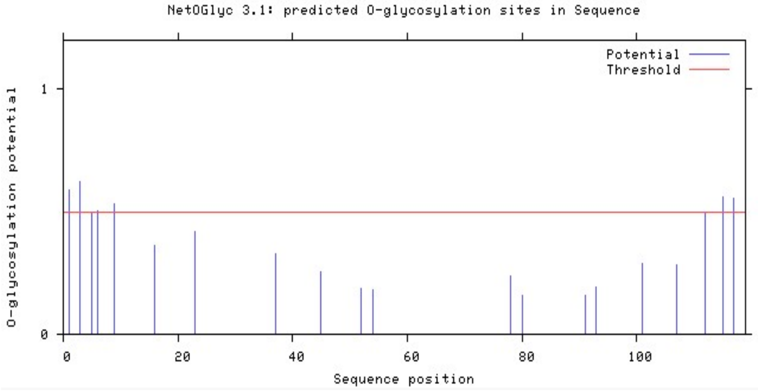

(**B**)

Figure S3. Multiple alignment of 1-119 amino acids sequences (without 19 amino acids signal peptide) in N-terminal protein of pSN, cSN and 6 types of mSN with PDB: 1OD9, 1URL, 1QFOa,b, 2BVEa,b as homology modeling template. (**A**) Multiple sequence alignment for pSN homology modeling; (**B**) Multiple sequence alignment for cSN homology modeling.

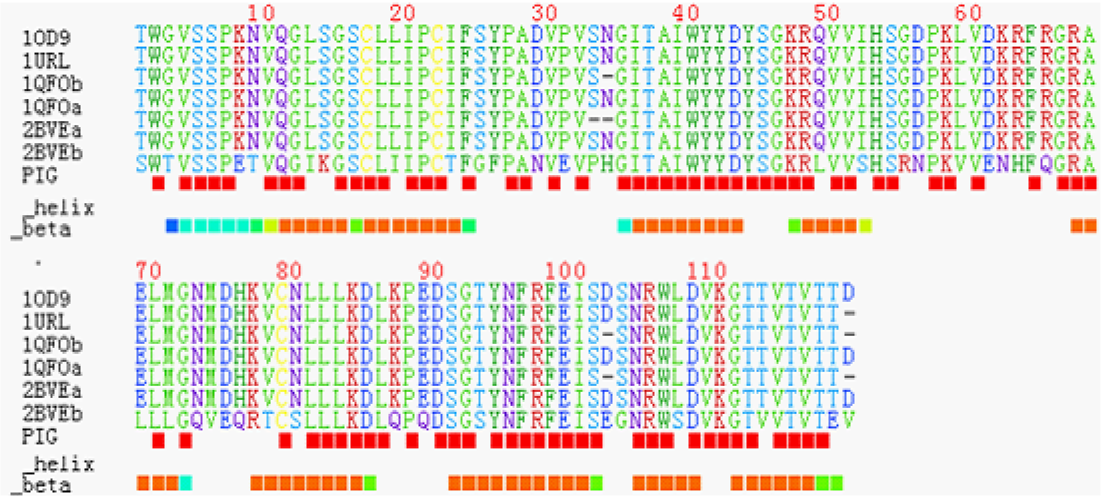

(**A**)

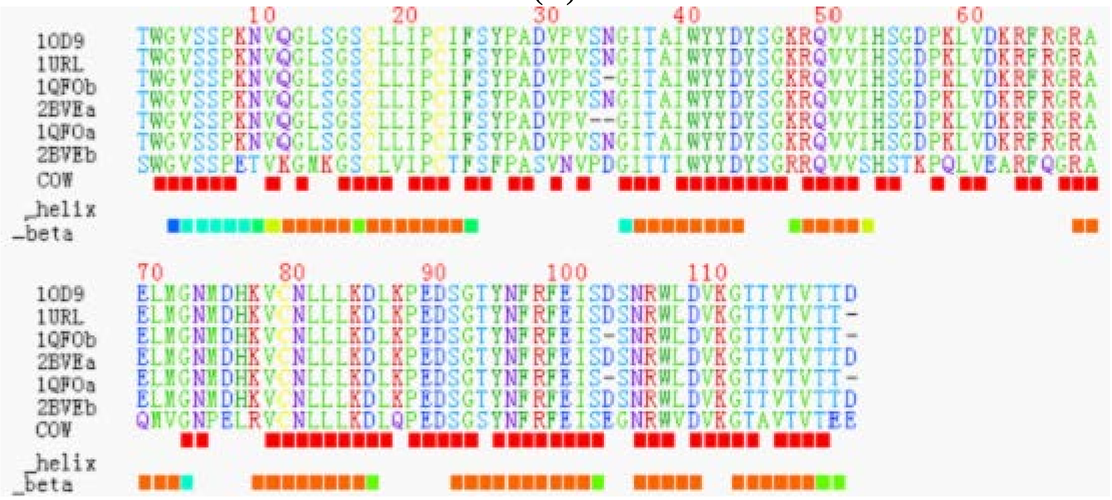

(**B**)

Figure S4. The protein MD (molecular dynamics) simulation of cSN and pSN. All the MD process lasted until 1440PS. Total energy, potential energy became low and stable. RMSD (Root Mean Square Deviation) variety after 1000PS kept between 0.5Å. The stable structure for quality assessment, superposition and mutant analysis. (**a**) pSN total energy varied with time; (**b**) pSN potential energy varied with time; (**c**) pSN backbone RMSD varied with time; (**d**) cSN total energy varied with time; (**e**) cSN potential energy varied with time; (**f**) cSN backbone RMSD varied with time.

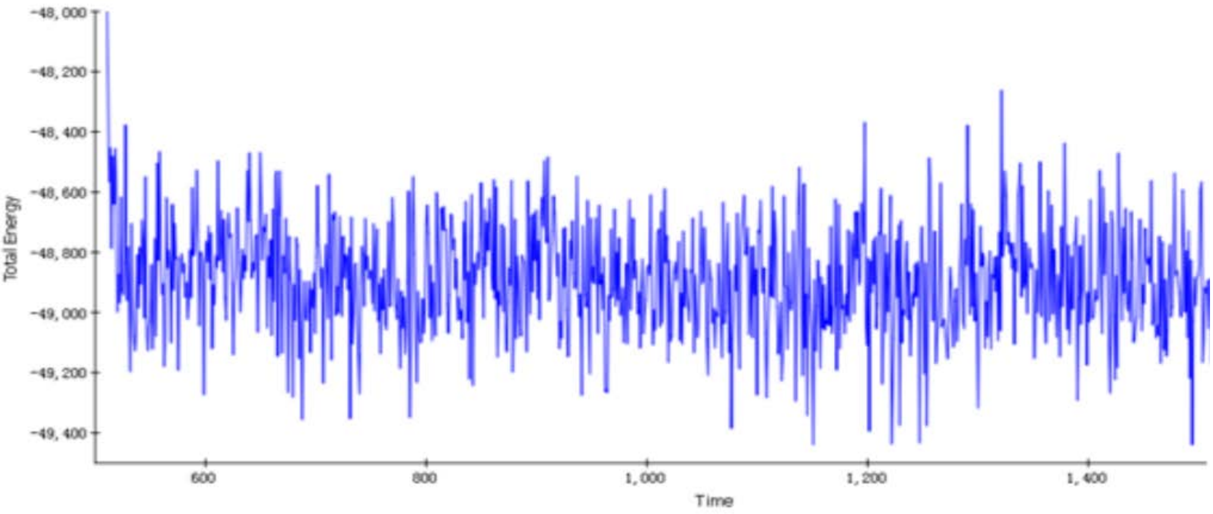

(**A**)

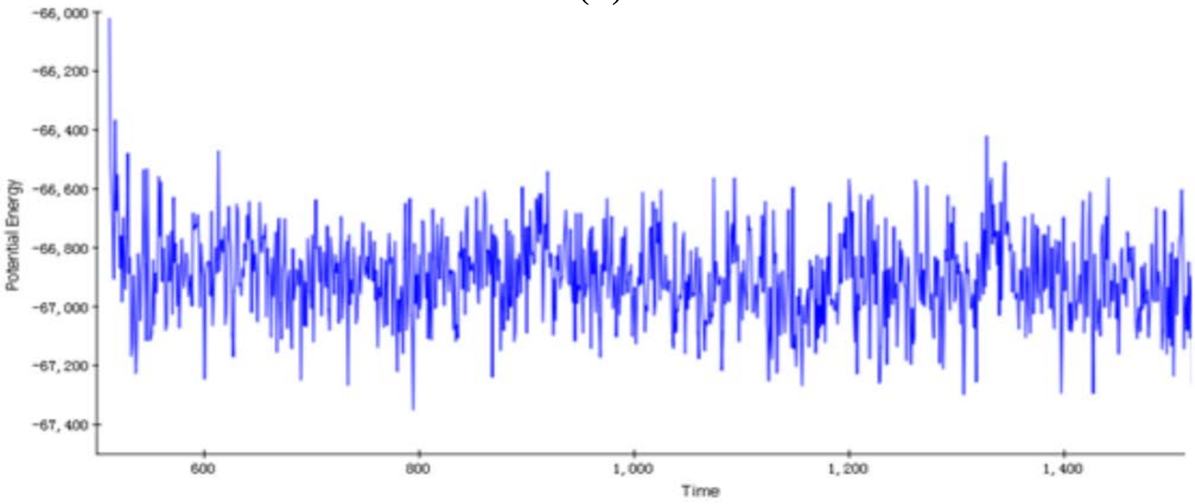

(**B**)

Figure S4. *Cont.*

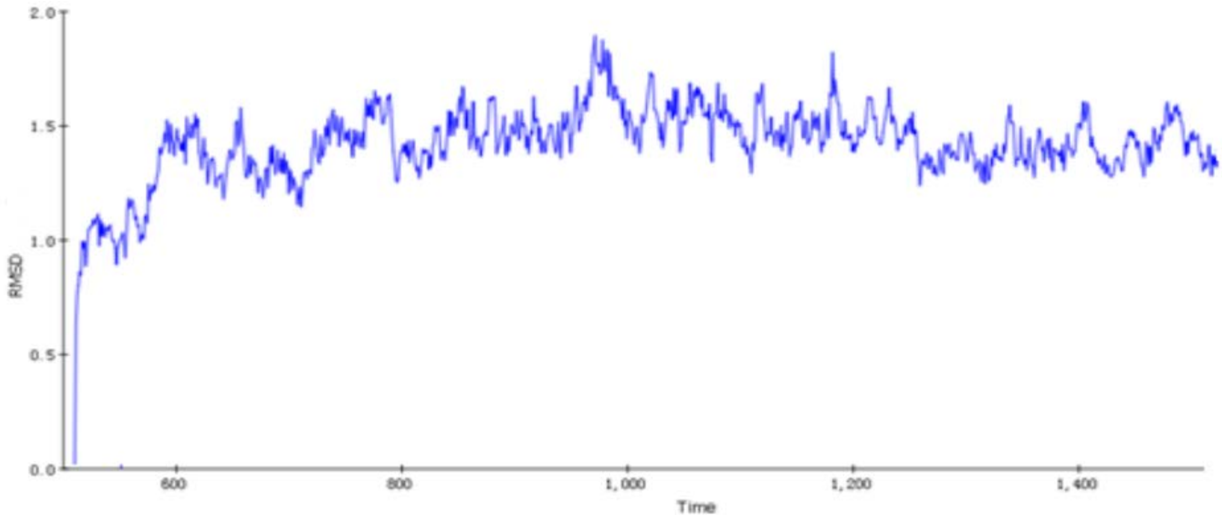

(**C**)

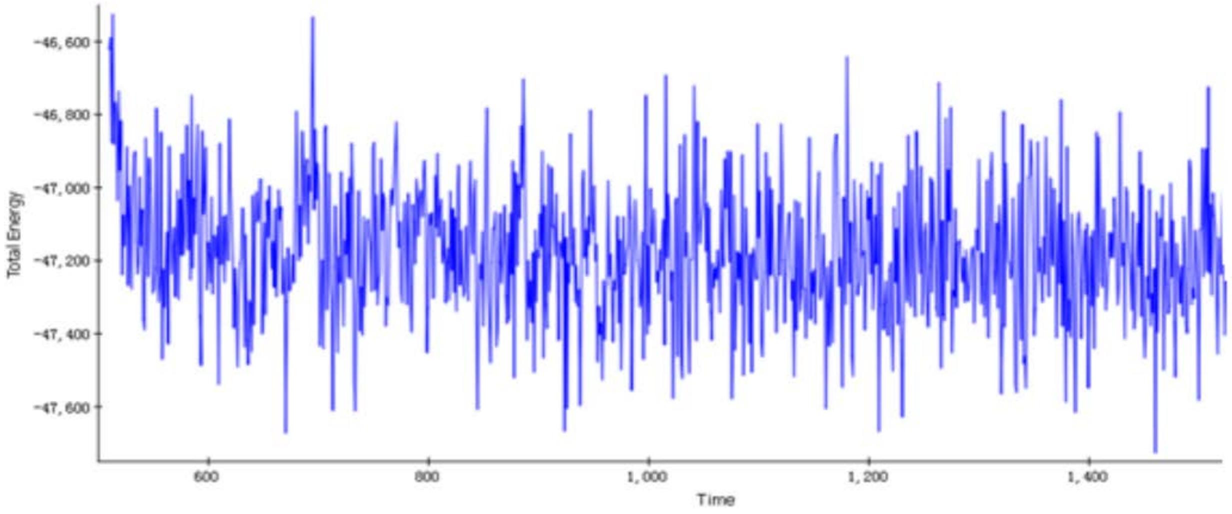

(**D**)

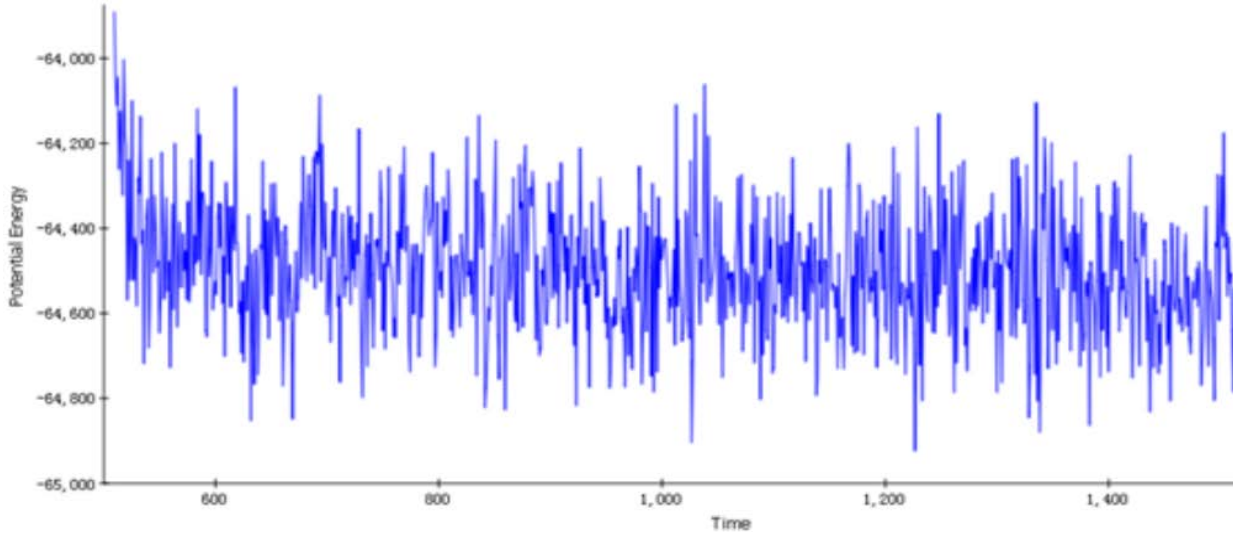

(**E**)

Figure S4. *Cont.*

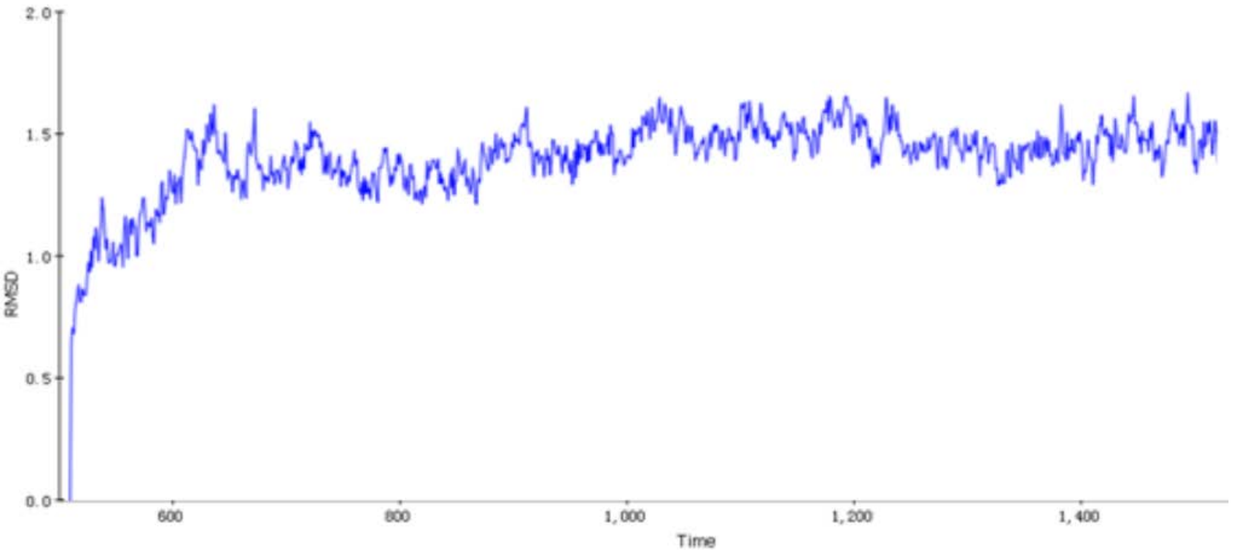

(**F**)

© 2013 by the authors; licensee MDPI, Basel, Switzerland. This article is an open access article distributed under the terms and conditions of the Creative Commons Attribution license (http://creativecommons.org/licenses/by/3.0/).
